# Operationalizing Population Salutogenesis Through Recovery-Oriented Cognitive Therapy: Evidence from Schizophrenia

**DOI:** 10.31083/AP48857

**Published:** 2026-03-17

**Authors:** Shin-ichiro Sasahara, Kei Muroi, Shun Nakajima

**Affiliations:** ^1^Institute of Medicine, University of Tsukuba, 305-8577 Tsukuba, Ibaraki, Japan; ^2^Research and Development Center for Lifestyle Innovation, University of Tsukuba, 305-8577 Tsukuba, Ibaraki, Japan; ^3^International Institute for Integrative Sleep Medicine (WPI-IIIS), Tsukuba Institute for Advanced Research (TIAR), University of Tsukuba, 305-8577 Tsukuba, Ibaraki, Japan

**Keywords:** schizophrenia, cognitive behavioral therapy, salutogenesis, sense of coherence, mental health recovery, community mental health services

Dear Editor,

Karaçam Doğan and Guloksuz [1] demonstrate how exposome-wide association studies (ExWAS) map modifiable environmental factors for population mental health interventions, aligning with the population salutogenesis paradigm, which emphasizes health promotion over disease treatment alone [2]. Although ExWAS identifies factors that can be targeted, a critical question remains: how can we systematically implement interventions that translate these insights into practice? We propose that Recovery-Oriented Cognitive Therapy (CT-R) integrated with salutogenic principles through the sense of coherence (SOC) provides an evidence-based framework for instituting population salutogenesis across multiple levels.

SOC—which perceives life as comprehensible, manageable, and meaningful—is central to Antonovsky’s theory of salutogenesis and strongly protects against mental health problems (r = –0.46 from childhood to young adulthood) [3], yet remains underutilized clinically. CT-R, developed by Grant *et al*. [4], initiates recovery by identifying aspirations, activating adaptive functioning, and fostering connections. Randomized trials demonstrate that CT-R improves functioning (effect sizes 0.52–0.68) and reduces negative symptoms in patients with schizophrenia [4]. Critically, CT-R has been implemented across individual therapy, group interventions, milieu programming, and community systems, underscoring its applicability from clinical to population levels.

**Fig. 1.**
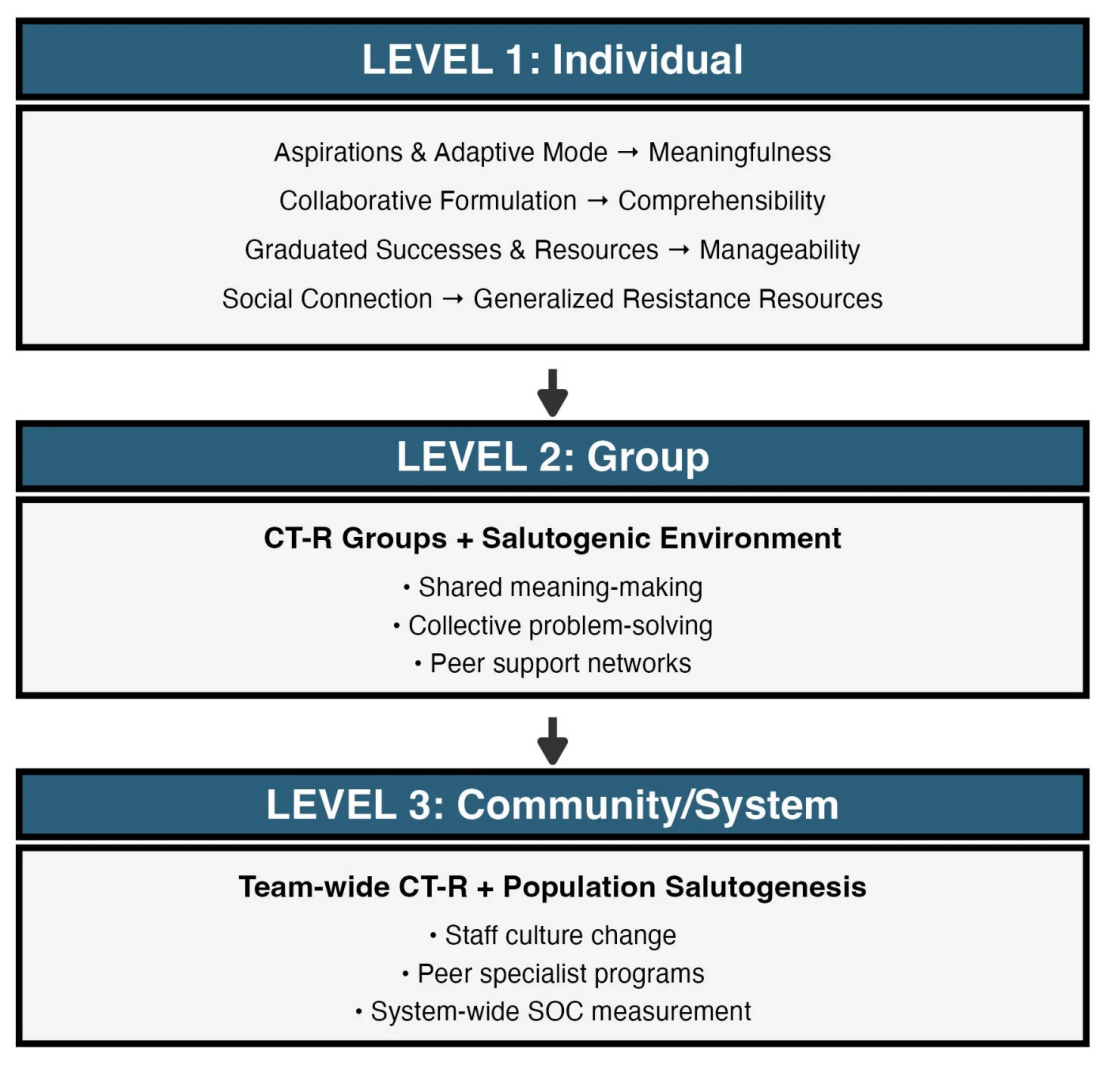
**Multilevel Integration Framework: SOC-Informed CT-R Across Individual, Group, and System Levels**. The framework illustrates three integrated levels of implementation for SOC-informed CT-R. Level 1 (Individual) shows how CT-R components map onto SOC dimensions: aspirations and adaptive mode enhance meaningfulness; collaborative formulation builds comprehensibility; graduated successes and resource mobilization increase manageability; and social connection aligns with generalized resistance resources. Level 2 (Group) demonstrates how CT-R groups create salutogenic environments through shared meaning-making, collective problem-solving, and peer support networks. Level 3 (Community/System) depicts system-wide CT-R implementation combined with population salutogenesis principles, including staff culture change, peer specialist programs, and system-wide SOC measurement. This vertical integration enables systematic scaling from evidence-based individual therapy to community-wide health promotion. CT-R, Recovery-Oriented Cognitive Therapy; SOC, Sense of Coherence.

We propose a multilevel, SOC-informed CT-R to bridge the gap between clinical care and population health. To our knowledge, this is the first theoretical framework to explicitly integrate these approaches across the individual, group, and system levels (Fig. 1).

At Level 1 (Individual Therapy), CT-R components correspond directly to SOC dimensions: aspirations and adaptive mode activation strengthen meaningfulness; collaborative formulation enhances comprehensibility; graduated successes and resource mobilization increase manageability; and social connection aligns with generalized resistance resources. When a person expresses helplessness regarding their capabilities, CT-R therapists collaboratively explore aspirations (meaningfulness), reframe beliefs (comprehensibility), identify resources and past successes (manageability), and strengthen therapeutic rapport. This systematically builds the SOC while addressing negative symptoms.

Schizophrenia is a critical test for population salutogenesis. Despite adequate pharmacological treatment, persistent negative symptoms, cognitive deficits, and functional impairments in schizophrenia exemplify the need for interventions that enhance comprehensibility, manageability, and meaningfulness. Cognitive symptoms, including deficits in attention, working memory, executive function, and cognitive flexibility, represent significant obstacles to recovery and are closely linked to SOC dimensions [5]. Recent evidence demonstrates that cognitive flexibility and well-being collectively account for 63% of the variance in SOC scores among patients with schizophrenia, with significant positive correlations across all dimensions [5]. This suggests that interventions simultaneously addressing cognitive deficits and SOC building may synergistically enhance recovery. CT-R addresses these cognitive challenges through structured problem-solving, graduated task engagement, and cognitive restructuring, thereby helping to compensate for cognitive limitations while building functional capacity and a sense of coherence. In addition, core mechanisms addressed by CT-R (defeatist performance beliefs, social withdrawal, and meaning-making deficits) extend beyond schizophrenia to mood disorders, anxiety disorders, and severe personality pathology, suggesting a broader transdiagnostic applicability of the SOC-informed framework.

At Level 2 (Group and Milieu Interventions), group CT-R facilitates shared meaning-making, collective problem-solving, and peer support. Inpatient milieu programs using CT-R principles have reduced coercive interventions and improved the unit climate [6]. The Inte.G.R.O. program, which combined salutogenic psychoeducation with a group format, produced sustained functional improvements over 36 months in patients with severe mental illness, demonstrating how group-based salutogenic interventions translate individual gains into broader social functioning [7].

At Level 3 (System and Community Implementation), CT-R has been implemented across community teams and state systems, shifting the organizational culture from deficit-focused crisis management to strength-based health promotion. Peer specialists trained in CT-R principles facilitate community-based SOC groups, extending professional interventions into natural settings and bridging clinical care with the population health approach advocated by Karaçam Doğan and Guloksuz [1]. Barriers to implementation can be addressed through brief training modules, peer champions, and pilot demonstrations.

The proposed integration addresses a key gap in population salutogenesis, moving from the identification of modifiable exposures to the implementation of scalable interventions. ExWAS can identify targets, such as social isolation or childhood adversity, and SOC-informed CT-R provides an intervention methodology to address these factors simultaneously at the individual, group, and system levels. Preliminary evidence suggests that SOC is a viable treatment target, as it has been shown to strongly mediate the effects of mindfulness-based CBT on the quality of life [8].

Key research questions include whether SOC mediates CT-R effects on functioning, whether multilevel interventions provide incremental benefits, and whether system-level implementation shifts the distribution of the SOC population. Pragmatic trials comparing standard and SOC-informed CT-R could quantify this added value.

Aaron Beck operationalized coherence-building clinically through Recovery-oriented Cognitive Therapy’s emphasis on aspirations, adaptive functioning, and meaningful connection. Aaron Antonovsky articulated coherence as a population construct through salutogenesis. Karaçam Doğan and Guloksuz [1] have concluded that, “a transformative impact on population mental health… only requires a comprehensive approach”. Multilevel, SOC-informed CT-R offers one such comprehensive approach—using existing evidence-based interventions systematically scaled through salutogenic principles.
